# A cardiologic approach to non-insulin antidiabetic pharmacotherapy in patients with heart disease

**DOI:** 10.1186/1475-2840-8-38

**Published:** 2009-07-20

**Authors:** Enrique Z Fisman, Alexander Tenenbaum

**Affiliations:** 1 Sackler Faculty of Medicine, Tel-Aviv University, 69978 Ramat-Aviv, Tel-Aviv, Israel; 2 Cardiovascular Diabetology Research Foundation, 58484 Holon, Israel; 3 Cardiac Rehabilitation Institute, Sheba Medical Center, 52621 Tel-Hashomer, Israel

## Abstract

Classical non-insulin antihyperglycemic drugs currently approved for the treatment of type 2 diabetes mellitus (T2DM) comprise five groups: biguanides, sulfonylureas, meglitinides, glitazones and alpha-glucosidase inhibitors. Novel compounds are represented by the incretin mimetic drugs like glucagon like peptide-1 (GLP-1), the dipeptidyl peptidase 4 (DPP-4) inhibitors, dual peroxisome proliferator-activated receptors (PPAR) agonists (glitazars) and amylin mimetic drugs. We review the cardiovascular effects of these drugs in an attempt to improve knowledge regarding their potential risks when treating T2DM in cardiac patients. Metformin may lead to lethal lactic acidosis, especially in patients with clinical conditions that predispose to this complication, such as recent myocardial infarction, heart or renal failure. Sulfonylureas exert their effect by closing the ATP-dependent potassium channels. This prevents the opening of these channels during myocardial ischemia, impeding the necessary hyperpolarization that protects the cell. The combined sulfonylurea/metformin therapy reveals additive effects on mortality in patients with coronary artery disease (CAD). Meglitinides effects are similar to those of sulfonylureas, due to their almost analogous mechanism of action. Glitazones lower leptin levels, leading to weight gain and are unsafe in NYHA class III or IV. The long-term effects of alpha-glucosidase inhibitors on morbidity and mortality rates is yet unknown. The incretin GLP-1 is associated with reductions in body weight and appears to present positive inotropic effects. DPP-4 inhibitors influences on the cardiovascular system seem to be neutral and patients do not gain weight. The future of glitazars is presently uncertain following concerns about their safety. The amylin mimetic drug paramlintide, while a satisfactory adjuvant medication in insulin-dependent diabetes, is unlikely to play a major role in the management of T2DM.

Summarizing the present information it can be stated that 1. Four out the five classical oral antidiabetic drug groups present proven or potential cardiac hazards; 2. These hazards are not mere 'side effects', but biochemical phenomena which are deeply rooted in the drugs' mechanism of action; 3. Current data indicate that the combined glibenclamide/metformin therapy seems to present special risk and should be avoided in the long-term management of T2DM with proven CAD; 4. Glitazones should be avoided in patients with overt heart failure; 5, The novel incretin mimetic drugs and DPP-4 inhibitors – while usually inadequate as monotherapy – appear to be satisfactory adjuvant drugs due to the lack of known undesirable cardiovascular effects; 6. Customized antihyperglycemic pharmacological approaches should be implemented for the achievement of optimal treatment of T2DM patients with heart disease. In this context, it should be carefully taken into consideration whether the leading clinical status is CAD or heart failure.

## Introduction

Diabetes mellitus threatens to become a global health crisis; treating diabetes and its complications is going to dominate future health care expenditures. Type 2 diabetes mellitus (T2DM) accounts for about 90% of the total diabetic population, and coronary artery disease (CAD) is the most common cause of morbidity and mortality. Cardiovascular deaths are increased up to fourfold in diabetics compared with their nondiabetic counterparts [1]. More than two-thirds of people with diabetes are obese. They require drugs that stimulate beta-cells to make more insulin and/or drugs that help insulin work better. When these do not work any longer, people require insulin. Unfortunately this form of diabetes is growing at an alarming rate. Since these patients will receive antidiabetic therapy indefinitely, any undesirable cardiovascular effects from well-known and widely used oral antidiabetic drugs should be analyzed in depth. In patients with T2DM, the University Group Diabetes Program (UGDP) reported in 1970 a higher frequency of major cardiovascular events in patients treated with tolbutamide, a sulfonylurea [2]. Awareness of this issue has increased during recent years following the detection of harmful influences of sulfonylureas on the ischemic myocardial cell [3,4]. On the other hand, cardiovascular derangement associated with the use of metformin has also been reported during both short [5,6] and long-term follow-up [7].

When oral antidiabetic monotherapy does not achieve the glycemic goal, combination treatment is implemented. A sulfonylurea – usually glibenclamide (known also as glyburide in the USA) – plus metformin constitute the most widely used antihyperglycemic combination in clinical practice [8]. However, the safety of this therapeutic regimen in long-term treatment is questionable [9]. The use of insulin in T2DM is also controversial. Nonetheless, after some years of disease oral therapy will be not yet effective and the majority of patients will receive insulin [10]. The issue whether the adverse cardiovascular effects of several medications may be additive and detrimental for the cardiac patients is of paramount importance and has not yet been specifically addressed in problem-oriented studies.

Insulin resistance represents the background of a series of common factors for the development of both diabetes and heart disease. These factors include genetics, hypertension, obesity, hyperglycemia, dyslipidemia, prothrombotic state, aging, physical inactivity. Once both diseases are clinically established, antidiabetic therapy *per se *may lead to a further derangement of cardiovascular status. Five types of classical oral antihperglycemic drugs are currently approved for the treatment of diabetes: biguanides, sulfonylureas, meglitinides, glitazones and alpha-glucosidase inhibitors. The novel antihyperglycemic compounds are represented by the incretin mimetic drugs, the dipeptidyl peptidase (DPP-4) inhibitors, the dual peroxisome proliferator-activated receptors (PPAR) agonists (glitazars) and the amylin mimetic drugs.

We will briefly review the cardiovascular effects of the most commonly used antidiabetic drugs within these types, in an attempt to improve knowledge and awareness regarding their influences and potential risks when treating cardiac patients.

## Classical medications

### Biguanides

The biguanides were launched in the 1950's, and metformin is the only drug belonging to this class currently available in most parts of the world. It reduces blood glucose levels through suppression of gluconeogenesis, stimulation of peripheral glucose uptake by tissue (mainly skeletal muscles) in the presence of insulin, and decreased absorption of glucose from the gastrointestinal tract. It has no direct effects on beta-cells, does not produce hypoglycemia, reduces glycohemoglobin (HgA1c) and improves both blood lipid profile and fibrinolytic activity. In contrast to other antidiabetic medications, metformin does not cause weight gain and appears to be the drug of choice in obese patients.

Despite these beneficial effects, metformin presents potential disadvantages that may influence the cardiovascular system. Gastrointestinal disturbances such as diarrhea are frequent, and the intestinal absorption of group B vitamins and folate is impaired during chronic therapy [11]. This deficiency may lead to increased plasma homocysteine levels which, in turn, accelerate the progression of vascular disease due to adverse effects on platelets, clotting factors, and endothelium. The existence of a graded association between homocysteine levels and overall mortality in patients with CAD is well established [12]. In addition, metformin may lead to lethal lactic acidosis, especially in patients with clinical conditions that predispose to this complication, such as heart failure or recent myocardial infarction [6]. It should be remembered that other drugs of the biguanide group – phenformin and buformin – were withdrawn in many countries during the 1970's due to its link with lactic acidosis. A possible association of phenformin with increased cardiovascular mortality has also been suggested [13]. Finally, metformin undergoes renal excretion, presenting undesirable pharmacologic interactions with several widely used cardiovascular drugs. The coadministration of nifedipine or furosemide leads to increased metformin plasma levels. Furthermore, digoxin, quinidine, and triamterene – which are eliminated by renal tubular secretion – may interact with metformin by competing for proximal renal tubular transport systems [14]. Metformin was introduced in the USA in 1995, and serious controversies regarding cardiovascular safety followed its approval for use [5]. We have found increased mortality in CAD patients receiving metformin after a 5-year follow-up [7]. However, it should be stressed that this finding ought be treated with caution since it arose from a nonrandomized study in which information on drug doses and severity and duration of diabetes was incomplete. Moreover, in further studies metformin therapy was found to be associated with a favorable cardiac outcome. Compared to sulfonylureas, lesser morbidity in patients with heart failure [15] and lesser cardiovascular hospitalization and mortality were reported [16].

### Sulfonylureas

These compounds are available nearly half a century. Today, sulfonylureas continue to represent a mainstay of therapy in patients with T2DM; their hypoglycemic potency is directly related to baseline plasma glucose values [17]. At the cellular level, they exert their action by closing the ATP-dependent potassium channels; this feature is responsible for both the insulinotropic effect and the adverse effects on the heart [3,4]. Namely, sulfonylureas bind with high affinity to a subunit of these channels leading to depolarization of the cell. Under physiologic conditions, the channels remain closed. During ischemia, sulfonylureas may prevent their opening, avoiding the necessary hyperpolarization that protects the cell by impeding calcium influx [4]. In this context, it should be stressed that cardiac and vascular sulfonylurea receptors are structurally different from their pancreatic analog [4]. In fact, sulfonylureas have been reported to reduce resting myocardial blood flow [18] to impair the recovery of contractile function after experimental ischemia [19], to increase the ultimate infarct size [20], to elicit proarrhythmic effects [21], to abolish ischemic preconditioning in animal models [22], and to increase early mortality in patients with diabetes mellitus after direct angioplasty for acute myocardial infarction [23]. Deterrence of myocardial preconditioning by glibenclamide has also been demonstrated in clinical trials [24].

It is important to stress that not all the undesirable effects on cardiovascular outcome reported by the UGDP for the first-generation sulfonylureas such as tolbutamide [2] can be automatically extrapolated to the more modern second-generation compounds such as glibenclamide or gliplizde [3]. In our experience, cardiovascular mortality rates in CAD patients on sulfonylureas (mainly glibenclamide) were lower than those on combined sulfonylurea-metformin therapy, and similar to the rates in patients on diet alone [7]. Another relatively new sulfonylurea, glimepiride, is more pancreas-specific and does not show interaction with cardiovascular ATP-dependent potassium channels [3,17,24].

### Megltinides

Meglitinides are insulin secretagogues. The first drug of this group, repaglinide, a benzoic acid derivative, was introduced in the USA in 1998. The second, nateglinide, is a d-phenylalanine derivative. Like sulfonylureas, these compounds act by closing the ATP-dependent potassium channels. However, its mechanism of action seem to be more complex since possibly three meglitinide receptor binding sites have been found on the beta-cells [25].

Despite a common basic mechanism of action, the insulinotropic effects of the two approved agents can be differently influenced by ambient glucose, leading to dissimilar responsiveness. Nateglinide may exert a more physiologic effect on insulin secretion – i.e. a glycemia-dependent response – than repaglinide, presenting less propensity to elicit hypoglycemia in vivo [26]. On the other hand, nateglinide presents a relatively lesser influence on HgA1c levels. When used as monotherapy, these drugs reduce both fasting plasma glucose and HgA1c, and have no significant effects on lipid profile. They present some specific characteristics that differentiate them from sulfonylureas: pills are taken before meals (the medication should not be administered if a meal is skipped), exhibit a short onset of action and a short pharmacologic half life, and act mainly on postprandial glucose.

The cardiovascular safety of these insulin secretagogues is still uncertain. Increased morbidity, particularly acute ischemic events, was observed for repaglinide after 1 year compared with glibenclamide. Nevertheless, patients on repaglinide appeared to have had more severe CAD at baseline than those in the glibenclamide group, and when adjustments were made the relative risk declined [27]. Thus, while definite assertions regarding cardiovascular safety cannot be made at this stage, caution should be implemented in view of the strong involvement of the ATP-dependent potassium channels in the mechanism of action.

Regarding nateglinide, it appears to have less affinity for the potassium channels than repaglinide [28] and it is interesting to mention a double effect: its action as a prandial insulin-releasing agent may partly rely on inhibition of GLP-1 degradation as well as beta-cell ATP-dependent potassium channels inhibition [29].

### Glitazones

This group of drugs, called also thiazolidinediones, was introduced in 1997 and includes antidiabetic medications such as troglitazone, pioglitazone, and rosiglitazone, the chemical structure and mechanism of action of which are very different from those of the other groups. Chemically, they are thiazolidinediones having chroman moieties; some of the analogues may present an aminoalkyl group as a linker between the chroman ring and the phenoxy moiety. Troglitazone, which was the first agent in this class to receive labeling approval, was withdrawn from clinical use in the US due to hepatotoxicity [30].

These drugs are insulin sensitizers, and they bind to the PPAR gamma, leading to increased glucose transporter expression. Sensitivity to insulin – especially in adipocytes, muscle and liver – is improved, and an additional major effect is the inhibition of hepatic gluconeogenesis [31]. It should be pointed out that no increment in insulin secretion is documented. PPARs are transcription factors belonging to the superfamily of nuclear receptors; three isoforms (alpha, beta/delta, gamma) are known nowadays, which regulate glucose homeostasis, lipoprotein metabolism, local immune responses, local inflammation, tumors development, thrombosis and present also potential antiatherogenic effects [32].

Rosilitazone monotherapy is only modestly effective in reducing glucose and HgA1c levels. Plasma triglycerides are reduced by 10–20%, and high density lipoprotein (HDL) cholesterol levels increase by 5–10%, since it also stimulates the isoform PPAR-alpha that regulates lipid metabolism. These favorable effects are counterbalanced by a 10–15% increase in low density lipoprotein (LDL) cholesterol [33]. Edema has been reported in 5% of patients, and glitazones are contraindicated in NYHA class III or IV [33]. Regarding hepatotoxicity, studies with rosiglitazone and pioglitazone indicate that it is not a class effect. Further differences in the safety profiles of these agents arise because the oxidative metabolism for each agent occurs by distinct cytochrome pathways: pioglitazone involves CYP 3A4 and CYP 2C8 whereas rosiglitazone is principally metabolized by CYP 2C8. CYP 3A4 is involved in the metabolism of over 150 drugs, hence the potential for drug interactions with pioglitazone is much greater than with rosiglitazone. Class effects include slight reductions in hemoglobin and hematocrit, due to hemodilution [30].

It was stressed that rosiglitazone reduces urinary albumin excretion in T2DM and may even mildly reduce blood pressure [34]. Nontraditional markers of cardiovascular disease – such as matrix metalloproteinase-9 – may be reduced as well [35]. Another notorious characteristic of glitazones is their capability of lowering leptin levels, leading to several degrees of weight gain, usually proportional to the administered dose [34]. This feature has obvious harmful clinical implications and was documented in both experimental [36,37] and human studies [34]. In addition, it was suggested that rosiglitazone may be associated with a significant increase in the risk of myocardial infarction and with an increase in the risk of death from cardiovascular causes that had borderline significance [38]. Since the publication of this meta-analysis, it has been debated whether rosiglitazone should remain on the market. To date, the US Food and Drug Administration (FDA) has concluded that there is insufficient evidence to withdraw rosiglitazone, yet questions about its cardiotoxicity persist. Most recently published studies in this issue demonstrated that rosiglitazone may be safer than previously thought: rosiglitazone does not increase the risk of overall cardiovascular morbidity or mortality compared with standard glucose-lowering drugs [39]. In addition, when added to metformin or a sulfonylurea, a 12-month treatment with rosiglitazone reduced ambulatory blood pressure to a greater extent than when metformin and a sulfonylurea are combined [40], confirming previous data regarding beneficial influence on blood pressure [34].

Pioglitazone treatment in patients with advanced T2DM at high risk for cardiovascular events participating in the PROactive Study, yielded significant risk reductions in major adverse events composite end points at 3 years [41,42]. The risk of heart failure is a class effect of the thiazolidinediones, whereas the ischemic cardiovascular risk is confined to rosiglitazone but not to pioglitazone. It seems that the differential effects of rosiglitazone and pioglitazone on metabolism may motivate the apparent disparity in their impact on outcomes: for example, pioglitazone lowers LDL cholesterol while rosiglitazone raises it [43].

Thus, glitazones exhibit a broad landscape of complex clinical effects, in part favorable and in part detrimental for the cardiovascular system. The concluding balance between these effects requires further elucidation. In this context, it should be mentioned that only few head-to-head comparisons of drug regimens containing glitazones versus drug regimens containing insulin were found [44]. This leaves clinicians unable to evaluate the effectiveness of one combination regimen over another. Many published large trials were composed by industry sponsored studies, increasing so the concern that funding source could influence outcomes and conclusions of the research [44].

### Alpha-glucosidase inhibitors

The primary mechanism of action of antidiabetic drugs like acarbose, voglibose and miglitol is grounded on competitive inhibition of several enzymes of the alpha-glucosidase group (maltase, isomaltase, sucrase, glucoamylase). These are membrane-bound enzymes that hydrolyze oligosaccharides and disaccharides to glucose in the brush border of the small intestine Thus, by delaying digestion of carbohydrates, these compounds shift their absorption to more distal parts of the small intestine and colon, and defer gastrointestinal absorption of glucose. Their hypoglycemic potency is less than that of biguanides and sulfonylureas [33] and, unlike the latter, they do not cause hypoglycemia. The most frequent side-effects to these drugs are mild abdominal pain, flatulence and diarrhea [45].

It is well established that impaired fasting glucose concentrations in nondiabetic patients with ischemic heart disease are a marker for a worse prognosis [46,47]. Acarbose could be used, either as an alternative or in addition to changes in lifestyle, to delay development of T2DM in these patients. Acarbose is by far the most extensively studied alpha-glucosidase inhibitor. The STOP-NIDDM trial [45] is the largest randomized trial to date investigating the drug in subjects with prediabetes and early diabetes. This study suggests that acarbose treatment was associated with a reduction in hypertension and cardiovascular disease: this treatment resulted in a 25% relative risk reduction in the development of T2DM, in a 34% risk reduction in the development of new cases of hypertension, and in a 49% risk reduction in cardiovascular events.

Interestingly, and additional metabolic pathway for these compounds was recently described. Chronic treatment with voglibose stimulates GLP-1 secretion and decreases plasma DPP-4 activity by reducing its circulating levels [48,49]. Similar features were documented for miglitol [50]. Thus, the antihyperglycemic effect of alpha-glucosidase inhibitors is likely achieved by two parallel mechanisms which are both direct and indirect. It directly reversibly inhibits the α-glucosidase enzymes, and secondly, induces GLP-1 secretion.

### Combined treatment with classical drugs

**Figure 1:**
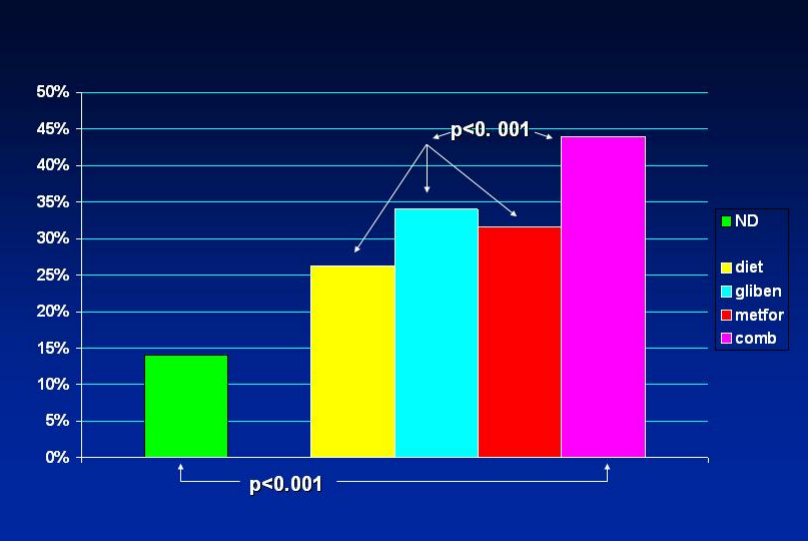
**Histographical display of crude all-cause mortality (percent) over a mean 7.7-year follow-up in 11,322 CAD patients – nondiabetic and diabetic – on several therapeutic regimens**. Mortality in patients on a combined glibenclamide/metformin regimen was significantly higher and almost quadrupled the figures documented for nondiabetic CAD patients. Significant statistical differences were still present when comparing the group on combined pharmacotherapy with the groups on other antidiabetic regimes. ND – nondiabetics; diet – patients solely on diet; gliben – patients on glibenclamide; metfor – patients on metformin; comb – patients on a combined glibenclamide/metformin regimen. (Based on data from Ref. [45]).

**Figure 2:**
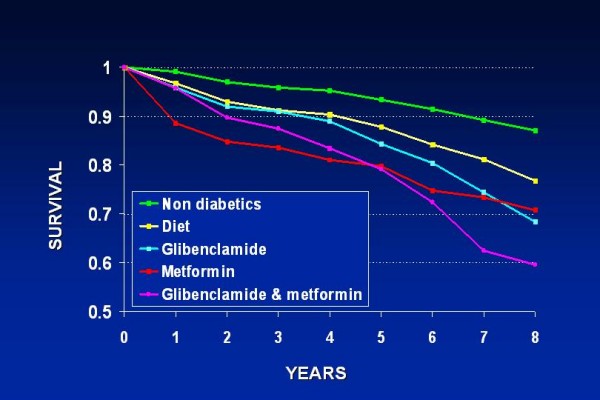
**Actuarial survival curves of all-cause mortality after multivariate analysis in the same population as in Figure 1**. Multivariate analysis included age, gender, glucose, cholesterol, triglycerides, previous myocardial infarction, anginal syndrome, hypertension, functional class, previous cerebrovascular accident, peripheral vascular disease, smoking, body mass index and use of beta blockers and antiplatelet drugs. With patients on diet as reference group, the combined treatment with metformin and glibenclamide was associated with a significantly higher hazard ratio of all cause mortality: 1.53 (95% CI 120–1.96. (Modified from Ref. [45]).

Combined therapy is based on the premise that pharmacological agents acting via different mechanisms and presenting differing side effects permit the design of individualized antidiabetic regimens. This approach reflects the plausibility that monotherapy with any currently available medication is likely to fail over time in some patients, and this type of pharmacological diabetes management is widely used. Findings from the United Kingdom Prospective Diabetes Study (UKPDS) showed that after 3 years, approximately 50% of patients could attain satisfactory glucose levels with monotherapy; by 9 years this had declined to only 25% [51]. Long-term problem-oriented prospective studies that focus specifically on the outcome of coronary diabetics on combined therapy are lacking. Data from an observational study – which included exclusively documented coronary patients – performed at our laboratory [52] indicated an all-cause increased crude mortality over a mean 7.7-year follow-up in diabetics on combined treatment with metformin and glibenclamide. Figures on mortality in this group almost quadrupled those of nondiabetic CAD patients (Figure 1). These results were corroborated when multivariate analysis was performed (Figure 2). Another study that focused on the general diabetic population found that there was an higher cardiovascular mortality in T2DM taking sulfonylurea and metformin in combination than in those taking only sulfonylurea [53], concluding that it cannot be excluded that this kind of combination therapy possibly increases cardiovascular mortality. Combination therapy is known to promote additional blood glucose reduction but there is as yet no evidence that these or another antidiabetic formulations are beneficial in preventing or delaying macrovascular disease. These observations are in keeping with the UKPDS reports demonstrating excess risk of all-cause mortality in the whole diabetic population receiving combined therapy, especially in patients in whom metformin was added at an early stage [54]. Likewise, a recent meta-analysis of observational studies [55] further confirms that the combination therapy of metformin and sulfonylurea significantly increases the relative risk of the composite end point of cardiovascular hospitalization or mortality (fatal and nonfatal events) irrespective of the reference group – diet therapy or metformin or sulfonylurea monotherapies.

Hence, the combined antihyperglycemic treatment with classical drugs leads to a peculiar entanglement since sulfonylureas and metformin are 1) the most powerful antiabetic drugs; 2) those presenting the most unfavorable cardiac effects; 3) the most frequently employed combination in routine clinical practice [56,57].

## New compounds

Taking into consideration the intimate interrelationship between diabetes and heart disease, the American Heart Association has coined in 1999 the phrase 'diabetes is a cardiovascular disease' [58]. Diabetic patients with CAD connote an enormous population which deserve a specific approach. Incretins, DPP-4 inhibitors, glitazars and the amylin mimetic drugs represent novel pharmacological approaches.

### Incretins

The progressive impairment of beta cell function and increased insulin demand as tissue becomes insulin resistant are core pathophysiologic defects in the development of hyperglycemia in T2DM [59,60]. Anyway, the process is a complex one and other important factors are also involved, further exacerbating the clinical setting. Excess glucagon secretion, abnormally accelerated gastric emptying during hyperglycemia, obesity, and increased food intake all contribute to hyperglycemia. Impaired release or action of incretin hormones, particularly GLP-1, and to a lesser degree glucose-dependent insulinotropic polypeptide (GIP), also play a role in the development and/or progression of T2DM. More recently, the essentially glucocentric view of the genesis and progression of T2DM has been supplemented by a more lipocentric perspective. Here the major mechanism is progressive ectopic lipid deposition (e.g., in myocytes and hepatocytes, rather than in adipocytes). Build-up of ectopic fat in those tissues ultimately induces insulin resistance, cell lipotoxicity, and diminished cell function, leading to metabolically inadequate insulin secretion [61].

Thus, incretin mimetic drugs are nowadays extensively investigated. A key role for intestinal peptides in the regulation of postprandial insulin secretion and glucose levels was proposed, based on the observation that insulin responses to an oral glucose load exceeded those measured after intravenous administration of an equivalent amount of glucose [62]. This phenomenon, termed the "incretin effect," postulated the existence of gut-derived signals promoting insulin secretion in response to nutrient intake [63]. Subsequently, the incretin hormones GIP and GLP-1, were discovered [64]. These two principal incretin hormones are small peptides – 42 and 30 amino acids, respectively, that rapidly stimulate the release of insulin only when blood glucose levels are elevated, thereby enhancing the glucose-sensing and insulin secretory capacity of the endocrine pancreas during postprandial hyperglycemia [65].

Physiological actions of incretins were extensively defined in animal studies with exogenous GLP-1 and GLP-1 receptor antagonists, highlighting its role as a meal-stimulated factor with potent glucose-lowering activity. Of significant clinical relevance is that exogenous GLP-1 has the potential to normalize fasting plasma glucose concentrations in patients with T2DM. In several studies in subjects with diabetes, GLP-1- whether administered by intravenous or subcutaneous infusion – normalized both fasting and postprandial glycemia by enhancing glucose-mediated insulin secretion, as well as by suppressing glucagon secretion [66-69].

Additional studies in animals and humans have demonstrated glucose-lowering effects of GLP-1. GLP-1 slows gastric emptying to decrease the rate of nutrient absorption, which results in more synchronous nutrient delivery with endogenous insulin action. Significant acute reductions in appetite and food intake after intravenous administration of GLP-1 in both healthy individuals and in patients with T2DM have also been demonstrated [70-72].

The mechanism through which the incretin hormones elicit their cytoprotective effects on the beta cell has attracted significant attention because preservation and restoration of beta-cell mass may contribute to the therapeutic potential of the incretins for the treatment of both type 1 and T2DM. Endoplasmic reticulum stress within the beta cell, possibly occurring as the result of the overproduction or misfolding of insulin, may be a contributing factor to the increased beta-cell apoptosis and loss of islet mass observed in diabetic patients [73].

Exenatide was first in the new class of incretin mimetics for the treatment of patients with T2DM. Several short-term phase 2 clinical trials have reported that subcutaneos exenatide acutely lowered both fasting and postprandial plasma glucose concentrations. The rate of gastric emptying was also slowed in patients treated with exenatide. Large-scale clinical trials designed to assess the safety and efficacy of twice daily subcutaneos exenatide over a six-month period were completed in subjects with T2DM who were unable to attain glycemic control with oral sulfonylureas, metformin, or both [74]. A novel formulation of exenatide consisting of biodegradable polymeric microspheres that entrap exenatide and provide extended release enabling once-weekly administration was recently developed [75]. It resulted in significantly greater improvements in glycemic control than exenatide given twice a day, with no increased risk of hypoglycemia and similar reductions in bodyweight [76].

Another incretin mimetic compound, liraglutide, is a once-daily GLP-1 derivative in development for the treatment of T2DM. GLP-1, in its natural form, is short-lived in the body (the half-life after subcutaneous injection is approximately 1 hour), so it is not very useful *per se *as a therapeutic agent. However, prolonged activity is achieved by chemical manipulation, reaching to 11–15 hours and making it suitable for once-daily dosing. This is attained by attaching a fatty acid molecule at one position of the GLP-1 molecule, enabling it to bind to albumin within the subcutaneous tissue and bloodstream. The active GLP-1 is then released from albumin at a slow, consistent rate. Binding with albumin also results in slower degradation and reduced elimination of liraglutide from the circulation by the kidneys compared to its natural form [77,78]. A recent report demonstrated that liraglutide once a day provided significantly greater improvements in glycemic control than did exenatide twice a day, and was generally better tolerated. The results suggest that liraglutide might be a treatment option for T2DM, especially when weight loss and risk of hypoglycemia are major considerations [79].

Additional long-acting incretin mimetic drugs, like albiglutide and taspoglutide are currently under investigation, presenting encouraging results [80,81].

### Dipeptidyl peptidase-4 inhibitors

While discovered in 1967, serine protease DPP-4 was only subject of intensive research during recent years. DPP-IV is ubiquitously expressed and exhibits postproline or alanine peptidase activity, thereby generating biologically inactive peptides via cleavage at the N-terminal region after *X*-proline or *X*-alanine. It is a complex molecule that exists as a membrane-spanning cell-anchored protein that is expressed on many cell types, and as a soluble form in the circulation; both forms have proteolytic activity.

Because both GLP-1 and GIP have an alanine residue at position 2, they are substrates for DPP-4. DPP-4 inhibitors like sitagliptin are orally administered drugs that improve glycemic control by preventing the rapid degradation of incretin hormones, thereby resulting in postprandial increases in levels of biologically active intact GLP-1 and GIP [82,83].

Sitagliptin is an orally-bioavailable selective DPP-4 inhibitor – the first one approved by the FDA – that was discovered through the optimization of a class of beta-aminoacid-derived DPP-4 inhibitors. It lowers DPP-4 activity in a sustained manner following once daily administration, preserves the circulating levels of intact GIP and GLP1 following meals in both acute and chronic studies and reduces blood glucose levels without significant increases in hypoglycemia [84]. Thus, the drug works by inhibiting the inactivation of the incretin GLP-1 and GIP by DPP-4. By preventing GLP-1 and GIP inactivation, GLP-1 and GIP are able to potentiate the secretion of insulin and suppress the release of glucagon by the pancreas. As the blood glucose level approaches normal, the amounts of insulin released and glucagon suppressed diminishes thus tending to prevent an overshoot and subsequent hypoglycemia which is seen with some other oral hypoglycemic agents.

Several additional DPP4 inhibitors, like saxagliptin, vildagliptin and alogliptin are currently investigated. Vildagliptin is the second DPP-4 inhibitor approved in Europe. Similarly to sitagliptin, vildagliptin has pharmacokinetic properties that support a once daily dosing regimen. Alogliptin in combination with pioglitazone, in an experimental model, improves glycemic control, lipid profiles, and increases pancreatic insulin content [85]. Whereas hepatic insufficiency does not seem to alter pharmacokinetics of these compounds, dose adjustments are required in patients with renal impairment, at least for sitagliptin [86]. Their beneficial effects are 1. increase circulating levels of GLP-1 in animals and humans; 2. increase the genesis, proliferation and differentiation of beta cells; 3. inhibit apoptosis of these cells; 4. enhance insulin secretion; 5. reduce fasting glucose; 6. reduce postparandial glucose; 7. reduce HbA1c levels.

In comparison to DPP-4 inhibitors, incretin mimetic agents have more pharmacological specificity but require subcutaneous injections. As with DPP-4 inhibitors, improvements in glycemic control were achieved with either no weight gain or with weight loss. Considering the impact of obesity on diabetes, along with weight gain that generally accompanies the use of insulin, insulin secretagogues, and insulin sensitizers, interventions with favorable effects on weight are likely to become increasingly important.

It should be pinpointed that there is a risk of potential adverse effects of DPP-4 inhibitors, especially on the immune system: an increased relative risk of 34% for all-cause infections after sitagliptin treatment was observed. Although, the risk of increased infection appears small, its consequences when translated into clinical practice with millions of T2DM patients treated could be considerable [87]. Thus, while DPP-4 inhibitors present some advantages over other antidiabetic agents, long-term data on potential cardiovascular effects are needed before widespread use of these drugs is recommended.

### Dual and pan-PPAR agonists

There are three PPARs subtypes which are commonly designated PPAR alpha, PPAR gamma and PPAR beta/delta. PPAR alpha activation increases HDL cholesterol synthesis, stimulates "reverse" cholesterol transport and reduces triglycerides. PPAR gamma activation results in insulin sensitization and antidiabetic action. Until recently, the biological role of PPAR beta/delta remained unclear. However, treatment of obese animals by specific PPAR delta agonists results in normalization of metabolic parameters and reduction of adiposity. Combined treatments with PPAR gamma and alpha agonists may potentially improve insulin resistance and alleviate atherogenic dyslipidemia, whereas PPAR delta properties may prevent the development of overweight which typically accompanies "pure" PPAR gamma ligands. Clearly, an optimal PPAR agent with improved safety profile that provides both effective glycemic and lipid control is needed. Compounds that affect both PPAR-alpha and PPAR-gamma, particularly with an optimized balance of agonist activity, might prove especially beneficial for patients with T2DM [88].

The old and well known lipid-lowering fibric acid derivative bezafibrate is the first clinically tested pan-(alpha, beta/delta, gamma) PPAR activator. It is the only pan-PPAR activator with more than a quarter of a century of therapeutic experience with a good safety profile. Therefore, bezafibrate could be considered (indeed, as a "post hoc" understanding) as an "archetype" of a clinically tested pan-PPAR ligand. Bezafibrate leads to considerable raising of HDL cholesterol and reduces triglycerides, improves insulin sensitivity and reduces blood glucose level, significantly lowering the incidence of cardiovascular events and new diabetes in patients with features of the metabolic syndrome. It attenuates the progression of insulin resistance, defers the onset of overt T2DM, enhances adiponectin levels and reduces the incidence of myocardial infarction in patients with metabolic syndrome during long-term follow-up [89-95]. However, from a biochemical point of view, bezafibrate is a PPAR ligand with a relatively low potency.

Several novel and potent dual PPAR-alpha/gamma agonists (glitazars) have been clinically developed. These agents have a major effect on peripheral and hepatic insulin sensitivity, with HbA1c reductions of 0.5–2%. On the basis of their mode of action, it is expected that these agents could modulate cardiovascular risk by improving endothelial reactivity, reducing blood pressure, and improving lipid profiles [96]. However, the emergence of different types of toxic effects in clinical trials has resulted in their failure to progress beyond phase III development. Nonetheless, no consistent safety signal has been detected, probably because PPAR-alpha and PPAR-gamma each control the expression of many proteins that are involved in a range of biological processes. For example, development of tesaglitazar was discontinued because of indications that it could cause renal impairment, muraglitazar was linked with cardiovascular safety issues, and the earlier agents ragaglitazar and farglitazar failed because of liver toxicity and tumors in rodents.

The recently reported SYNCHRONY study [97] aimed to establish the safety profile and glucose-lowering and lipid-modifying effects of the new compound aleglitazar [98]. Aleglitazar significantly reduced baseline HbA1c versus placebo in a dose-dependent manner, with a 600 μg dose. Edema, hemodilution, and weight gain occurred and were dose-dependent. However, at aleglitazar doses less than 300 μg, no patients had congestive heart failure, frequency of edema was similar to placebo and less than with pioglitazone, and bodyweight gain was less than with pioglitazone. The favorable balance in the safety and efficacy profile of aleglitazar represents encouraging short-term clinical data for this agent and provides good evidence to enter phase III investigation. Thus, the future of these dual PPAR agonists is presently uncertain following former concerns about their safety. However, the favorable changes in lipids and glycemic endpoints still encourage further research.

### Amylin mimetic drugs

Amylin is a synergistic partner to insulin, with which it is cosecreted from pancreatic beta cells in response to meals. Deficient amylin secretion is a well-recognized phenomenon in type 1 diabetes and in a later-stage in T2DM, in whom pancreatic insulin production is markedly reduced. Its physiological effects mimic in part those of GLP-1. Amylin suppresses glucagon – a pancreatic hormone that regulates the production of glucose by the liver – secretion from pancreatic alpha cells, thereby attenuating hepatic glucose production. It also delays gastric emptying and likely possesses a central effect to enhance satiety [99].

Pramlintide is a synthetic hormone for parenteral (subcutaneous) administration, resembling human amylin effects. It reduces the production of glucose by the liver by inhibiting the action of glucagon and diminishes postprandial glucose fluctuations. The drug was approved by the FDA in March 2005. While it seems to be a satisfactory adjuvant medication in insulin-dependent diabetes, it is unlikely to play a major future role in the management of T2DM [100].

### Cardiovascular effects of the new compounds

While he main physiologic benefits demonstrated from exenatide therapy have been on indexes of glycemic control, cardiovascular effects have also been described. In experimental models, GLP-1 receptors have been demonstrated in cardiac myocytes and in certain regions of the brain that regulate autonomic function [101]. In some cases the use of GLP-1 infusion was associated with a doubling of stroke volume and an increase in cardiac output by > 50%, as well as significant decreases in left ventricular end-diastolic volume [102]. Encouraging results were also documented in humans [103], but long-term safety data are required before we fully understand any potential benefits or risks derived from the hemodynamic influences of GLP-1-based therapies.

Regarding DPP-4 inhibitors, few data are available concerning cardiovascular markers or clinical outcomes. Given the preliminary data they might be considered in individuals with impaired ventricular function. However, no clinical trials using these agents have yet been reported in this or any other group of patients with cardiovascular disease. Glitazars may yield some reduction in blood pressure [96]. The amylin mimetic drug pramlintide did not show cardiovascular advantages or risks [99].

## Clinical implications

Our pharmacological armamentarium is nowadays increasingly complex, offering a wide array of drugs, both as monotherapy or in combination. It is therefore frequently difficult to determine the best therapeutic option for a given patient. A common problem arises when a drug is known to give a prompt and beneficial effect in the short term, but data regarding long-term outcome and safety are either lacking or insufficient. This is particularly true regarding antihyperglycemic drugs in patients with CAD.

Comprehensive risk reduction is mandatory for diabetic patients with CAD. General measures should comprise diet, physical activity, complete cessation of smoking, and weight and lipid profile management. However, fewer than 10% of patients achieve acceptable long-term glycemic values with non-pharmacological therapy only [104]. Special emphasis should be given to blood pressure control; we have reported the presence of widespread undiagnosed hypertension in this population, which presented a 5-year mortality even higher than that in diabetics previously identified as hypertensives [105]. Moreover, the increased mortality associated with hypertension in mild diet-treated type 2 diabetes strongly supports the need for early onset of antihypertensive treatment in these patients [106]. When examining the status of glucose metabolism in patients with heart failure secondary to CAD, it is disclosed that both T2DM and impaired fasting glucose are associated with increased prevalence of heart failure among patients with CAD [107].

Evidence is available that long-term maintenance of normal or near-normal glucose levels using pharmacological means is protective in diabetic patients, improving microvascular disease (retinopathy, nephropathy, and neuropathy) and reducing both morbidity and mortality [108,109]. Is this also applicable to coronary diabetics? Current data indicate that the answer is positive, but alleviation of macrovascular complications remains dubious. Moreover, the therapeutic criteria for T2DM patients without proven heart disease should not be automatically extrapolated to cardiac diabetic patients, who need a carefully customized treatment. The pathogenesis of atherosclerosis spans decades. Thus, there is an emerging notion that tight glycemic control may be beneficial in primary prevention of cardiovascular disease in younger patients with T2DM, but may become deleterious in older patients with established disease. Then, while a tight glycemic control may lessen microvascular disease, it may increase the risk for adverse cardiovascular events [110].

What should the policy be regarding the widely used sulfonylurea-metformin combined treatment? Following approval of a given therapy for a chronic condition, large prospective, randomized, placebo-controlled trials designed to check its long-term safety and effectiveness require many years to be completed, and sometimes such studies are not performed at all. This is the case with this combined treatment in CAD patients. The data from observational studies available at present indicate increased mortality in patients receiving this therapy [52-55] suggesting that this combination should be used with caution in diabetics with proven CAD. The excessive mortality rate could reflect an additive expression of the adverse cardiovascular effects of each of these medications. However, we would like to stress that our own observations specifically address the glibenclamide-metformin combined treatment [52] and we have no information regarding combinations of metformin with other sulfonylureas, such as glimepiride or gliclazide.

## Conclusion

Based on current data, stringent guidelines or strategies regarding non-insulin antidiabetic pharmacotherapy in T2DM patients with heart disease cannot yet be outlined. Anyhow, summarizing the available information it can be stated that

1. Four out the five classical oral antidiabetic drug groups present proven or potential cardiac hazards.

2. These hazards are not mere 'side effects', but biochemical phenomena which are deeply rooted in the drugs' mechanism of action.

3. Current data indicate that the combined glibenclamide/metformin therapy seems to present special risk and should be avoided in the long-term management of type 2 diabetics with proven CAD.

4. Glitazones should be avoided in patients with overt heart failure.

5, The novel incretin mimetic drugs and DPP-4 inhibitors – while usually relatively ineffective as monotherapy – appear to be satisfactory adjuvant drugs due to the lack of known undesirable cardiovascular effects.

6. Customized antihyperglycemic pharmacological approaches should be implemented for the achievement of optimal treatment of T2DM with heart disease. In this context, it should be carefully taken into consideration whether the leading clinical status is CAD or heart failure.

## Abbreviations

CAD: coronary artery disease; DPP-4: dipeptidyl peptidase 4; FDA: US Food and Drug Administration; GIP: glucose-dependent insulinotropic polypeptide; GLP-1: glucagon like peptide-1; HbA1c: glycohemoglobin; HDL: high density lipoprotein; LDL: low density lipoprotein; PPAR: peroxisome proliferator-activated receptor; T2DM: type 2 diabetes mellitus; UGDP: University Group Diabetes Program; UKPDS: United Kingdom Prospective Diabetes Study.

## Competing interests

The authors declare that they have no competing interests.

## Authors' contributions

Both authors have equally contributed in the conception and drafting of the manuscript.
